# Biosynthesis of long-chain omega-3 fatty acids by the nestlings of a generalist seabird

**DOI:** 10.1242/jeb.250429

**Published:** 2025-12-02

**Authors:** Jessika Lamarre, David R. Wilson

**Affiliations:** ^1^ Cognitive and Behavioural Ecology Program, Memorial University of Newfoundland and Labrador, St John's, Canada, A1B 3X9; ^2^ Department of Psychology, Memorial University of Newfoundland and Labrador, St John's, Canada, A1B 3X9

**Keywords:** Essential fatty acids, Bioconversion, Sprecher pathway, Marine environments, Urbanization, Gulls

## Abstract

Docosahexaenoic acid (DHA), a long-chain omega-3 fatty acid (n3-LCPUFA), is an integral component of vertebrate brains. Vertebrates maintain their DHA levels through biosynthesis using alpha-linolenic acid (ALA; omega-3 precursor) or by consuming preformed DHA and other n3-LCPUFAs which abound in the natural diets of marine predators. Yet, numerous marine predators, including generalist seabirds, now exploit anthropogenic resources potentially deficient in n3-LCPUFAs. Whether they can offset such deficiency by bioconverting ALA into DHA remains unknown. Here, we tested whether chicks of the ring-billed gull (*Larus delawarensis*), a generalist seabird thriving in cities, can biosynthesize n3-LCPUFAs, including DHA, from ALA. We brought into captivity 12 hatchlings from an urban colony and 12 from a natural colony. Nine hatchlings per colony were gavaged 490 μl of ALA-rich flaxseed oil daily for 3 days. The control groups (*N*=3 urban hatchlings, 3 natural hatchlings) received an omega-3-free caloric equivalent in place of the ALA supplement. All chicks received an omega-3-free diet throughout captivity (72 h). We also attempted to follow ALA's potential bioconversion into n3-LCPUFAs using an oral ^13^C_1_-enriched ALA tracer. Unfortunately, compound-specific isotope analyses of brain and liver tissue failed to detect any ^13^C enrichment. Nevertheless, the flaxseed oil supplementation study provided evidence of some ALA bioconversion. Compared with controls, supplemented chicks from both colonies accumulated more of all ALA derivates in their tissues except for DHA. We demonstrate for the first time that a seabird shows incomplete omega-3 bioconversion abilities, leaving them potentially vulnerable to deficiencies associated with urban foraging and shifting marine ecosystems.

## Teaser

**Summary:** Chicks of the ring-billed gull, a generalist seabird, can bioconvert the omega-3 precursor alpha-linolenic acid only into some of its longer-chain derivates, thus showing an incomplete ability to meet its metabolic requirements endogenously.

## INTRODUCTION

Omega-3 polyunsaturated fatty acids (n3-PUFAs) are nutrients essential to vertebrates (Castro et al., 2016). They are defined by their carbon chain containing multiple double bonds, one of which is situated on the third carbon from the methyl end of the chain. Alpha-linolenic acid (ALA; 18:3n3) is the shortest n3-PUFA, with 18 carbons and three double bonds, and is used as the precursor of long-chain polyunsaturated omega-3 fatty acids (n3-LCPUFAs; ≥20-long carbon chain; Sprecher, 2000). The n3-LCPUFAs eicosapentaenoic acid (EPA; 20:5n3), docosapentaenoic acid (DPA; 22:5n3) and docosahexaenoic acid (DHA; 22:6n3) have critical metabolic and structural roles in the tissues of vertebrates (Castro et al., 2016; Pilecky et al., 2021). DHA is the most important fatty acid in cerebral tissues because it promotes neurogenesis and is the main structural PUFA in neuronal membranes (Dyall, 2015; Janssen and Kiliaan, 2014; Roy et al., 2020; Speake and Wood, 2005). High levels of DHA in neuronal membranes help facilitate signal transduction (Janssen and Kiliaan, 2014; Lauritzen et al., 2016) and preserve life-long neuroplasticity (Dyall et al., 2010; Weiser et al., 2016), in addition to preserving membrane integrity by promoting an increase in antioxidant activity (Bazan, 2009; Bazinet and Layé, 2014). Accordingly, higher levels of cerebral DHA sustain optimized cognitive functions, including improved memory skills, executive functioning and learning abilities (e.g. Brenna and Carlson, 2014; Dyall, 2015; Lim et al., 2005; McNamara et al., 2010; Sasaki et al., 2024). EPA and DPA are used either as precursors of DHA or for the anti-inflammatory properties of their metabolites that help maintain or optimize the health and function of tissues found primarily within the nervous system (Bazinet and Layé, 2014; Calder, 2015; Liu et al., 2021; Yalagala et al., 2019). To meet their anatomical and physiological needs, vertebrates obtain these n3-LCPUFAs through their diet, by biosynthesizing them from the ALA precursor, or both (Bauer et al., 2014; Calder, 2015; Pilecky et al., 2021).

**Fig. 1.**
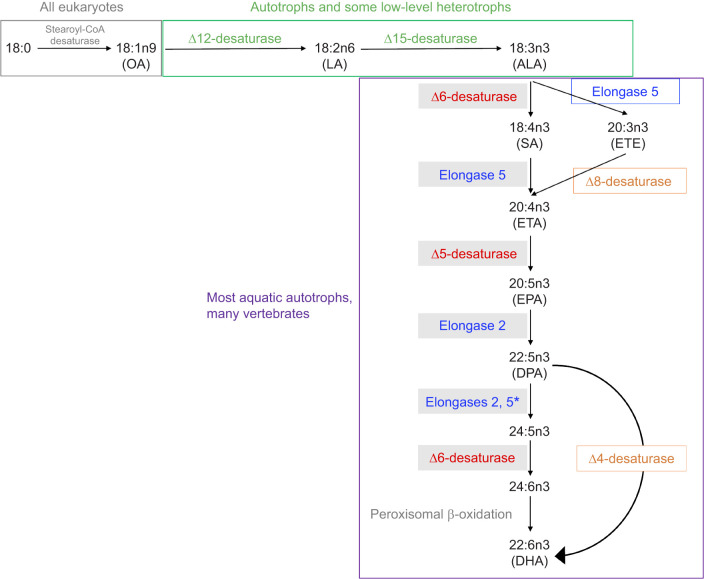
**Enzymatic pathway of the bioconversion of omega-3 polyunsaturated fatty acids (n3-PUFAs) in vertebrates.** Eicosapentaenoic acid (EPA), docosapentaenoic acid (DPA) and docosahexaenoic acid (DHA) are the main bioactive metabolites derived from alpha-linolenic acid (ALA). The shorter-chain omega-3 intermediates include stearidonic acid (SA), eicosatrienoic acid (ETE) and eicosatetraenoic acid (ETA). The precursors of ALA include stearic acid (18:0), which can be converted into oleic acid (OA) via the stearoyl-CoA desaturases found in all eukaryotes (grey lettering; Twining et al., 2021a). OA is converted into LA and ALA in autotrophs and some low-level heterotrophs that express Δ12- and Δ15-desaturases (green lettering; Garrido et al., 2019; Kabeya et al., 2018, 2020). The main n3-PUFA enzymatic pathway expressed by vertebrates (Sprecher pathway) is shaded and requires the action of elongases 2 and 5 (colored blue, present in all chordates; Monroig et al., 2016) and of Δ5- and Δ6- desaturases (colored red, absent in certain carnivorous vertebrates; Twining et al., 2021a). Although other enzymatic pathways exist, namely through Δ8- and Δ4-desaturases (colored orange), they account for minor bioconversion of their respective substrates in vertebrates (Monroig et al., 2011; Park et al., 2009, 2015). *Pathway found only in chicken (Gregory et al., 2013).

The biosynthesis of n3-PUFAs happens through an enzymatic pathway in which various elongases and desaturases work sequentially to introduce additional carbons and double bonds into the fatty acid chain (Fig. 1; Harwood, 1988). Vertebrates lack the enzymes needed to synthesize ALA *de novo* and therefore must obtain ALA or its derivatives from their diet (Tocher, 2015; Twining et al., 2021a). ALA is produced mainly by terrestrial autotrophs expressing the Δ12- and Δ15-desaturases needed to convert oleic acid (OA; 18:1n9) into linoleic acid (LA; 18:2n6) and LA into ALA (Fig. 1; Harwood, 1988). However, most vascular terrestrial plants lack the enzymes required to extend ALA into n3-LCPUFAs (Damude and Kinney, 2008; Napier and Graham, 2010). As a result, n3-LCPUFAs are limited in terrestrial ecosystems whereas ALA is abundant, particularly in wild land plants and in the invertebrates that feed on such plants (Gladyshev et al., 2016; Parmar et al., 2022; Twining et al., 2021a). Conversely, many aquatic autotrophs express all of the enzymes necessary to produce LA and ALA *de novo* and to convert ALA into EPA, DPA and DHA (Harwood and Guschina, 2009). Moreover, these aquatic autotrophs are efficient at producing n3-LCPUFAs because they use the Δ4-desaturase pathway to desaturate DPA directly into DHA (Fig. 1; Napier, 2007). Consequently, n3-LCPUFAs are abundant in the tissues of aquatic autotrophs (Colombo et al., 2016; Gladyshev and Sushchik, 2019; Hixson et al., 2015). This is especially true for marine ecosystems, where, compared with freshwater ecosystems, autotrophs produce higher levels of n3-LCPUFAs as a possible mechanism for maintaining membrane fluidity in cold and saline conditions (Aussant et al., 2018; Guschina and Harwood, 2006; Yokochi et al., 1998; Zhu et al., 2007). Longer food chains in marine waters also provide more n3-LCPUFAs to higher trophic levels because the fatty acids bioaccumulate through zooplankton and forage fish (Barrett et al., 2007; Kainz et al., 2004; Parrish, 2013).

Terrestrial vertebrates that consume plants have little access to preformed n3-LCPUFAs and therefore must convert dietary ALA into EPA, DPA and DHA to meet their requirements (Gladyshev et al., 2016; Twining et al., 2019). The capacity to bioconvert ALA into n3-LCPUFAs involves specific elongases and desaturases, commonly elongases -2 and -5, and Δ5- and Δ6- desaturases that, collectively, are referred to as the Sprecher pathway (Fig. 1; Leonard et al., 2004; Sprecher, 2000). This bioconversion capacity has been demonstrated in diverse primary and secondary terrestrial consumers, including chickens (*Gallus gallus domesticus*; Aguillón-Páez et al., 2020; Cherian, 2015; Kartikasari et al., 2012), monk parrots (*Myiopsitta monachus*; Petzinger et al., 2014), blue tits (*Cyanistes caeruleus*; Twining et al., 2021b), zebra finches (*Taeniopygia guttata*; McCue et al., 2009), ostriches (*Struthio camelus var. domesticus*; Poławska et al., 2013), livestock (Ebrahimi et al., 2014; Kronberg et al., 2012; Moallem et al., 2015) and rabbits (*Oryctolagus cuniculus*; Ander et al., 2010). These lower-level consumers store endogenously synthesized n3-LCPUFAs in their tissues, making them available to their higher-order terrestrial predators (Colombo et al., 2016; Hixson et al., 2015).

The bioconversion of ALA is metabolically costly such that animals benefit from consuming preformed n3-LCPUFAs that can be readily incorporated into their tissues and metabolized (Monroig et al., 2010; Morais et al., 2009). As a result, it often has been hypothesized that animals with diets naturally rich in n3-LCPUFAs (i.e. higher-order predators and aquatic consumers) have lost or never developed the ability to bioconvert ALA into its long-chain derivates (Castro et al., 2012; Tocher, 2010). Accordingly, we know of various species that show insufficient conversion of the ALA precursor into n3-LCPUFAs, including cats (*Felis catus domesticus*; Rivers et al., 1975; Trevizan et al., 2012), carnivorous marine fish (Castro et al., 2012; Nyunoya et al., 2021; Tocher, 2010; Tu et al., 2013), soft-shelled turtles (*Pelodiscus sinensis*; Lin and Huang, 2007) and western sandpipers (*Calidris mauri*; Dick et al., 2024). As high-order predators, cats meet their needs by consuming the brains and livers of their terrestrial prey, which are rich in n3-LCPUFAs (Cordain et al., 2002; Crawford et al., 1976). Likewise, marine consumers such as sandpipers and carnivorous fish meet their needs by consuming diets rich in n3-LCPUFAs (Colombo et al., 2016; Gladyshev et al., 2016; Hixson et al., 2015).

Although many higher-order predators and aquatic consumers have lost the ability to bioconvert ALA into n3-LCPUFAs, others have retained the ability despite the associated energetic cost (Castro et al., 2016; Monroig et al., 2022). This ability appears particularly widespread among vertebrate primary consumers and predators that forage on freshwater diets rich in ALA and EPA but deficient in DHA (Hixson et al., 2015; Ishikawa et al., 2019). Among them, domestic ducks (*Anas platyrhynchos* f. dom.) stand out for their high efficiency at converting ALA into DHA (Shahid et al., 2019; Wang et al., 2022). Other aquatic or semi-aquatic predators that show this ability to some degree include several species of freshwater and salmonid fish (Hastings et al., 2004; Tocher, 2010), tree swallows (*Tachycineta bicolor*; Twining et al., 2016) and caimans (*Caiman latirostris*; Leiva et al., 2021). For these species, the ability to bioconvert ALA into n3-LCPUFAs buffers them from possible dietary shortages of n3-LCPUFAs (Tocher, 2010) and may be an evolutionary response to the lack of DHA in freshwater ecosystems. Indeed, several populations of marine fish that either lacked or showed poor conversion abilities but subsequently evolved or enhanced their capability to synthesize DHA from ALA were then able to colonize freshwater habitats (Ishikawa et al., 2019; Matsushita et al., 2020). Nonetheless, the ability of freshwater vertebrates to bioconvert ALA into DHA typically is insufficient to meet the animal's metabolic needs, forcing them to supplement their endogenous biosynthesis by consuming aquatic organisms containing preformed n3-LCPUFAs (Castro et al., 2012). Interestingly, terrestrial omnivores are in a similar situation. Some of these species can bioconvert ALA into DHA, but the biosynthesis is insufficient owing to suboptimal expression of Δ5- and/or Δ6-desaturases such that dietary n3-LCPUFAs are also required (Castro et al., 2012). For example, humans, rodents, dogs (*Canis familiaris*) and opposum (*Didelphis virginiana*) can bioconvert ALA into n3-LCPUFAs but still must regularly consume preformed n3-LCPUFAs to maintain optimal levels of DHA in their tissues (Castro et al., 2012; Harris et al., 2009; Plourde and Cunnane, 2007; Wyrostek et al., 2023).

The ability to synthesize n3-LCPUFAs *de novo* has only been tested in one species of marine-dwelling birds, the western sandpiper, which was shown to bioconvert ALA into small amounts of EPA and DPA but ultimately failed to significantly increase their tissue levels of DHA (Dick et al., 2024). This result matches the prevailing hypothesis that marine predators, including shorebirds such as western sandpipers and seabirds, have no or limited ability to bioconvert ALA given the abundance of preformed n3-LCPUFAs in their traditional diet. An inability to synthesize n3-LCPUFAs could lead to nutritional stress for many marine-dwelling birds, especially in generalist seabird species that have shifted their diet towards anthropogenic refuse deficient in n3-LCPUFAs (Langlois and Ratnayake, 2015; Simopoulos, 2002). An alternative hypothesis would be that generalist seabirds have retained some capacity to synthesize n3-LCPUFAs, which could have contributed to their success at exploiting anthropogenic landscapes and resources. Several species of gulls, for example, appear to thrive while foraging in cities and landfills when breeding (e.g. yellow-legged gulls, *Larus michahellis*: Arizaga et al., 2013, Duhem et al., 2008; herring gulls, *Larus argentatus*: Enners et al., 2018, Serré et al., 2022; lesser black-backed gulls, *Larus fuscus*: Spelt et al., 2019; glaucous gulls, *Larus glaucescens*: Weiser and Powell, 2010). Here, we used ring-billed gulls (*Larus delawarensis*), a generalist seabird that historically consumed fish and invertebrates but that now often thrives in urban centres by consuming refuse, to test the competing hypotheses that a marine predator has either lost or retained the ability to bioconvert ALA into DHA and other n3-LCPUFAs.

We focused this study on the bioconversion of ALA in newly hatched chicks because studies on poultry and mammals suggest that omega-3 metabolism peaks in early life, presumably because of the high demand for DHA during rapid brain development (Brenna et al., 2009; Kodas et al., 2002; Poureslami et al., 2010; Surugihalli et al., 2022). As they age, humans and rodents show decreasing conversion rates of ALA (Burdge and Calder, 2005; Carnielli et al., 2007; Lin et al., 2018; Uauy et al., 2000), with the notable exception of fertile females, which show higher conversion efficiency, likely associated with the demands related to gestation and lactation (Extier et al., 2010; Lin et al., 2016). It remains unknown whether other taxonomic groups follow the same patterns. We captured and brought into captivity nestlings hatched from an urban-foraging colony and a marine-foraging colony and fed them a baseline diet devoid of n3-PUFAs. Experimental chicks were supplemented with flaxseed oil rich in ALA whereas control chicks were fed a caloric equivalent devoid of ALA (Table 1). We then compared the cerebral and hepatic fatty acid profiles of the chicks to determine whether the experimental group showed increased levels of ALA-derivates compared with the control group, and whether the level of each omega-3 (mass percentage of total identified fatty acids) differed based on the chicks' colony of origin. We focused on the liver, which is the main site for n3-PUFA bioconversion in vertebrates, and on brain tissue, which accumulates the bulk of circulating DHA derived from the diet or produced endogenously in the liver (Ouellet et al., 2009; Voss et al., 1991). Simultaneously, we attempted to track the fate of dietary ALA in the chicks' tissues using compound-specific stable isotope analysis, similar to Twining et al. (2016, 2021b). To this end, hatchlings were dosed with a ^13^C_1_-enriched ALA tracer to characterize the carbon isotopic composition of their ALA-derivates to determine whether they had arisen from endogenous conversion. Unfortunately, the ^13^C_1_-enriched tracer could not be detected in the chicks' tissues. Here, we briefly present our methods and potential explanations for this failure to inform future studies.

**Table 1.** Fatty acid composition, expressed as the mass percentage of total identified fatty acids, of the flaxseed oil supplement, coconut oil supplement and baseline diet (equal mass earthworms, mealworms and egg white) given to ring-billed gull chicks in captivity

Fatty acid	Flaxseed oil (%)	Coconut oil (%)	Baseline diet (%)
C14:0	0.04	57.58	1.65
C14:1	Trace	Trace	0.08
C16:0	5.27	28.51	21.54
C16:1*n*−11	0.01	Trace	Trace
C16:1*n*−9	0.03	0.07	0.54
C16:1*n*−7	0.08	n.d.	3.58
C16:1*n*−5	Trace	Trace	0.04
C16:2*n*−6	Trace	Trace	0.05
C17:0	0.05	0.03	0.13
C16:3*n*−4	0.03	n.d.	0.07
C16:4*n*−3	Trace	Trace	0.01
C16:4*n*−1	0.01	n.d.	0.02
C18:0	1.72	10.73	5.05
C18:1*n*−11	1.80	n.d.	0.01
C18:1*n*−9	3.10	n.d.	45.05
C18:1*n*−7	n.d.	0.15	0.94
C18:1*n*−6	14.43	Trace	0.01
C18:1*n*−5	Trace	n.d.	0.03
C18:2*n*−6 (LA)	14.83	2.50	19.14
C18:2*n*−4	0.13	n.d.	0.01
C18:3*n*−4	0.25	n.d.	0.02
C18:3*n*−3 (ALA)	57.42	Trace	0.86
C18:4*n*−3 (SA)	0.02	Trace	n.d.
C18:4*n*−1	Trace	Trace	Trace
C20:0	0.13	0.28	0.20
C20:1*n*−11	0.01	0.08	0.21
C20:1*n*−9	0.10	Trace	0.10
C20:1*n*−7	Trace	Trace	0.01
C20:2*n*−6	0.04	Trace	0.06
C20:4*n*−6 (AA)	Trace	n.d.	0.30
C20:3*n*−3 (ETE)	0.04	Trace	Trace
C20:4*n*−3 (ETA)	Trace	Trace	n.d.
C20:5*n*−3 (EPA)	Trace	n.d.	n.d.
C22:0	0.12	Trace	0.04
C22:1*n*−9	Trace	Trace	n.d.
C22:1*n*−7	Trace	Trace	n.d.
C22:2*n*−6	Trace	Trace	Trace
C22:4*n*−6	0.01	n.d.	0.07
C22:3*n*−3	Trace	Trace	Trace
C22:5*n*−6	Trace	n.d.	0.04
C22:4*n*−3	0.01	Trace	Trace
C22:5*n*−3 (DPA)	Trace	Trace	n.d.
C24:0	0.06	Trace	0.02
C22:6*n*−3 (DHA)	Trace	n.d.	n.d.
C24:1	0.20	n.d.	Trace
Σ SFAs*	7.40	97.13	28.63
Σ MUFAs^‡^	19.76	0.32	50.59
Σ PUFAs^§^	72.82	2.51	20.66
Σ *n*6−PUFAs^¶^	14.88	2.50	19.60
Σ *n*3−PUFAs**	57.48	Trace	0.86
Σ *n*3−LCPUFAs^‡‡^	Trace	Trace	n.d.

*Sum of saturated fatty acids: C14:0+C16:0+C17:0+C18:0+C20:0+C22:0+C24:0.

^‡^Sum of monounsaturated fatty acids: C14:1+C16:1*n*−11+C16:1*n*−9+C16:1*n*−7+C16:1*n*−5+C18:1*n*−11+C18:1*n*−9+C18:1*n*−7+C18:1*n*−6+C18:1*n*−5+C20:1*n*−11+C20:1*n*−9+C20:1*n*−7+C22:1*n*−9+C22:1*n*−7+ C24:1.

^§^Sum of polyunsaturated fatty acids: C16:2*n*−6+C16:3*n*−4+C16:4*n*−3+C16:4*n*−1+C18:2*n*−6+C18:2*n*−4+C18:3*n*−4+C18:3*n*−3+C18:4*n*−3+C18:4*n*−1+C20:2*n*−6+C20:4*n*−6+C20:3*n*−3+C20:4*n*−3+C20:5*n*−3+C22:2*n*−6+C22:4*n*−6+C22:3*n*−3+C22:5*n*−6+C22:4*n*−3+C22:5*n*−3+C22:6*n*−3.

^¶^Sum of omega-6 polyunsaturated fatty acids: C18:2*n*−6+C20:2*n*−6+C20:4*n*−6+C22:4*n*−6+C22:5*n*−6.

**Sum of omega-3 polyunsaturated fatty acids: C18:3*n*−3+C18:4*n*−3+C20:3*n*−3+C20:4*n*−3+C20:5*n*−3+C22:5*n*−3+C22:6*n*−3.

^‡‡^Sum of omega-3 long chain polyunsaturated fatty acids: C20:5*n*−3+C22:5*n*−3+C22:6*n*−3.

Trace, fatty acid found to be below 0.01%; n.d., fatty acid not detected.

## MATERIALS AND METHODS

### Subjects

**Fig. 2.**
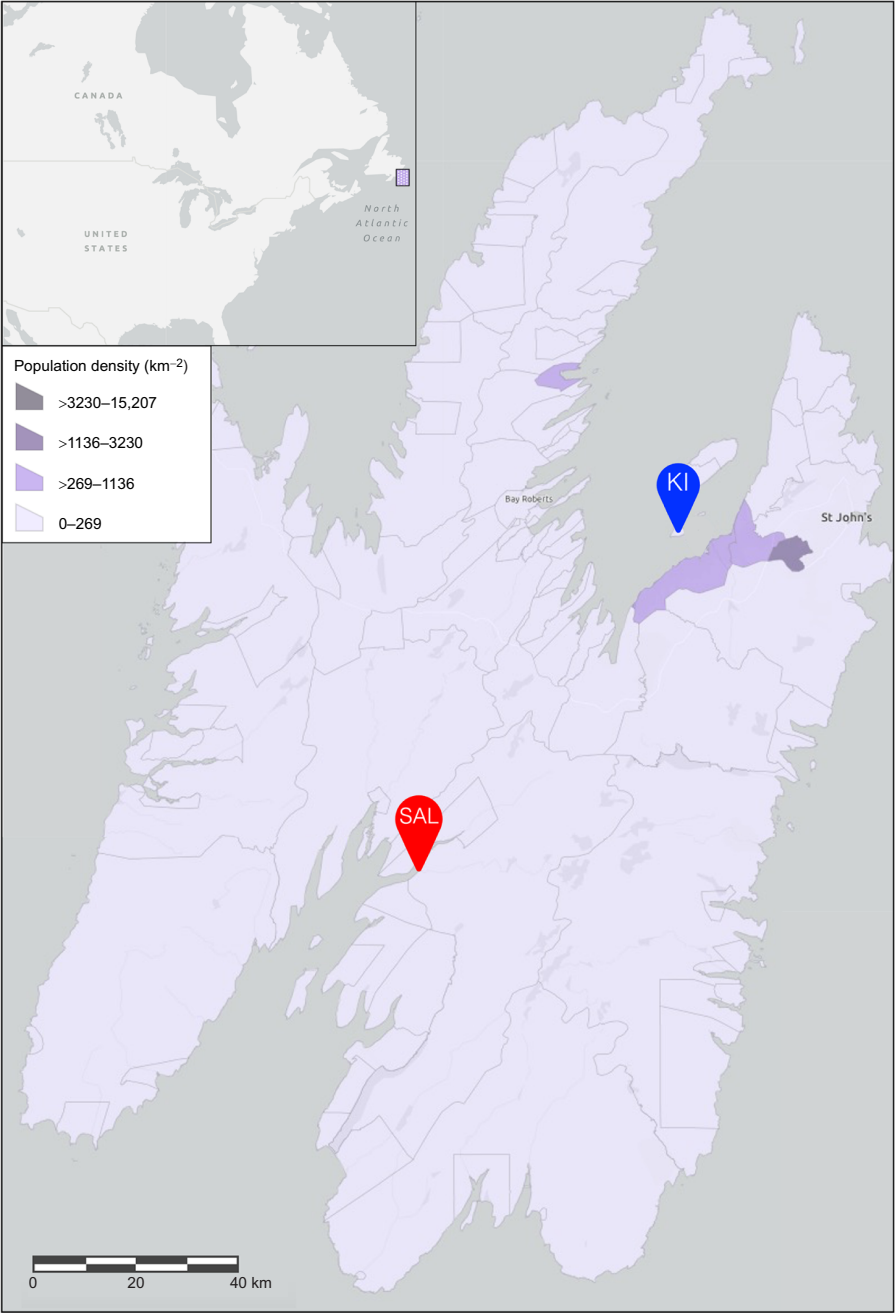
**Locations of the two colonies studied in Newfoundland, Canada, and the surrounding human population density (number of people per km^2^).** Census data are from 2021 (Esri Canada, https://www.arcgis.com/home/item.html?id=ee280506886549569a4c1955fe56916a). Kelly's Island (KI; 47°32′44.5″N 53°00′51.5″W) is an uninhabited island near an urban environment, whereas Salmonier (SAL; 47°08′11.0″N 53°28′48.6″W) is situated in a rural setting. The Salmonier colony is temporarily connected to the mainland by a sandbar at low tide (accessible on foot <5 h per day), whereas Kelly's Island is situated 2.7 km from the mainland. Both colonies are bordered by the Atlantic Ocean.

We monitored two breeding colonies of ring-billed gulls (*Larus delawarensis* Ord 1815) (Fig. 2) throughout their incubation period and then collected newly hatched chicks during peak hatching dates. We chose these colonies owing to known dietary differences between the gulls nesting at each site, with birds from Kelly's Island exploiting mainly anthropogenic foods deficient in n3-LCPUFAs and birds from Salmonier consuming mostly marine organisms rich in n3-LCPUFAs (Lamarre et al., 2022). Peak hatching at the Salmonier colony occurred on 18 June 2023. We collected the first hatchling from each of 12 three-egg clutches that still contained two unhatched eggs (either intact or pipping). Hatchlings were collected if they were wet or still had a protruding navel but no longer had a yolk sac attached; chicks remain in this condition for approximately 3 h post-hatch and typically are not yet fed by their parents (Kirkham and Morris, 1979; Tinbergen and Perdeck, 1950). We opted to use hatchlings rather than older chicks to control the fatty acid composition of all post-hatch feeds, in addition to preventing a delayed onset of first feed. Delayed first feed (>24–48 h) is associated with altered lipid synthesis and delayed yolk sac resorption (Wang et al., 2020b; Xu et al., 2022). In contrast, hatchlings fed early are expected to still be receiving some endogenous lipids from their remaining yolk sac, while also demonstrating the ability to absorb and potentially bioconvert dietary fatty acids (Mihelic et al., 2020; Noble and Cocchi, 1990; Speake and Deans, 2004). At Kelly's Island, we also collected the first hatched of 12 nests, although owing to the smaller size of the colony and the hatching asynchrony amongst nests, the chicks were collected over a 3-day period (21–23 June 2023) and came from either two- or three-egg clutches.

### Experimental procedure

In captivity, the hatchlings were housed together in a soft carrier lined with fleece and hot pads and kept indoors at 20°C. Each chick was uniquely identified with plastic tape attached to their leg and weighed using a digital scale (smart weigh TOP500; lot no. A14-336; accuracy: ±0.01 g) upon entering captivity and every 24 h subsequently. Immediately upon being brought into captivity, nine hatchlings from Salmonier and nine from Kelly's Island were assigned at random to the ALA experimental group and each was given an oral dose of 490 μl of flaxseed oil (Life brand™ Flaxseed Oil softgels, NPN: 80000193), which was repeated at 24 and 48 h of captivity. We chose this quantity of flaxseed oil as it replicates our methods from a previous study where ring-billed gull hatchlings received a dose of 490 μl of oil (fish oil), which represented half of their daily energetic requirement and was well tolerated by the subjects (Lamarre et al., 2021). Here, we used flaxseed oil as an ALA-rich supplement devoid of n3-LCPUFAs (Table 1) to test whether ring-billed gulls convert ALA into n3-LCPUFAs by comparing the concentration of ALA derivates (mass percentage of total identified fatty acids) present in the chicks' tissues based on their supplementation group. Per the manufacturer, 490 μl of flaxseed oil contains 234.8 mg of ALA. The control group received a caloric equivalent of coconut oil (Kirkland Signature™ Organic Virgin Coconut Oil; 490 μl) that is devoid of all n3-PUFAs.

We also attempted to track the bioconversion of ALA into its derivates by administering to the experimental chicks one prepared oral dose (1.36 mg) of an ALA-carbon isotope (ALA-^13^C_1_) tracer (Toronto Research Chemicals, cat. no. TRC-L467653, lot no. 4-SGP-68-2) dissolved in 100 mg of liquid coconut oil upon bringing them into captivity. The tracer has higher δ^13^C values than naturally occurring in n3-PUFAs, which can be used to follow the bioconversion of the labelled ALA into its derivates by examining the δ^13^C values of individual n3-PUFAs (Ali et al., 2025; Whiteman et al., 2019). Should ring-billed gulls show evidence of this bioconversion ability, each n3-PUFA derivate should present an artificially ^13^C-rich profile. We prepared the doses of ALA-^13^C_1_ in advance based on a similar study on blue tits (*Cyanistes caeruleus*) in which chicks were given approximately 0.035 mg of tracer per gram of body mass (Twining et al., 2021b). We used a mass of 39 g at hatch for first hatched ring-billed gull chicks based on previous work with this species (mean±s.d.: 39.03±4.26 g; range: 31.79 to 45.85 g; Lamarre et al., 2021). The body mass of the subjects collected here concurred with this estimate, with Salmonier chicks weighing 41.73±2.40 g (range: 37.65 to 46.06 g) and Kelly's Island chicks weighing 39.95±2.56 g (range: 36.15 to 44.20 g) at collection (Fig. S1). Therefore, each experimental subject was given a total dose of 1.36 mg of tracer dissolved in 100 μl of coconut oil (Kirkland Signature™ Organic Virgin Coconut Oil) to facilitate its administration. The remaining six chicks were assigned to the control group and only received the carrier coconut oil (100 μl) as a caloric equivalent devoid of n3-PUFAs (Table 1) and ^13^C_1_ tracer; this control group was used to establish the natural δ^13^C values of the chicks' fatty acid profiles. Unfortunately, we failed to detect the presence of the tracer in the chicks' tissues, likely owing to its insufficient enrichment as only the carbon from the carboxyl group of the ALA molecule was labelled with the ^13^C_1_ tracer.

The ALA-^13^C_1_ tracer, flaxseed oil and coconut oil were administered by gavage as this technique has been used without adverse effect to supplement 1-day-old ring-billed gulls with fish oil (Lamarre et al., 2021). To facilitate the digestion and absorption of the tracer and oil supplements, each gavage was immediately followed by a feed of their baseline diet. Chicks were hand-fed every 3 h during daylight hours (05:00 h to 20:00 h) throughout captivity by presenting them with food items containing no n3-PUFAs (equal weight earthworms, mealworms, cooked egg whites; Table 1). Each chick was fed this baseline diet individually until satiation. Seventy-two hours after the ALA-^13^C_1_ tracer or the caloric equivalent was administered, the hatchlings were euthanized by decapitation. We dissected out their cerebral hemispheres and liver, which were immediately placed at −20°C to await further analyses.

We chose these tissues for their respective functions and natural differences in accretion of various n3-PUFAs. The chicks' cerebral hemispheres were of particular interest given the rapid growth cerebral tissues undertake during early life, where rapid accretion of DHA and, to a lesser extent, DPA is expected (Drouin et al., 2019; Li et al., 2016; Speake and Wood, 2005). In contrast, other fatty acids, including ALA, have a low affinity for tissues rich in polar lipids such as the brain, resulting in their low concentration in cerebral hemispheres (Simopoulos, 2008). This contrasts with the liver, which is mainly composed of neutral lipids (Speake et al., 1996). As the main site for fatty acid metabolism (Meng et al., 2021; Valenzuela et al., 2024), the liver tends to store a certain level of ALA, which can then be converted into its various derivates, each stored in the liver until they are further metabolized or dispatched to other tissues (Gregory et al., 2011; Mashek, 2013).

All methods were performed under appropriate permits (Canadian Wildlife Service Scientific Permit, number SC4049; Environment and Climate Change Canada Scientific Permit to Capture and Band Migratory Birds, numbers 10890 and 10890B) and were approved by the Memorial University Animal Care Committee (number 22-03-DW).

### Fatty acid analysis

The flaxseed oil, coconut oil and baseline diet, as well as the brain and liver samples, were analyzed at the Core Research Equipment and Instrument Training Aquatic Research Cluster (CREAIT-ARC) facility at Memorial University. While frozen, each cerebral hemisphere and liver was homogenized using a grinder. We followed the methods of Folch et al. (1957), modified by using chloroform, methanol and chloroform-extracted water in a 2:1:0.5 ratio. We extracted the lipids from 30 mg of each homogenized cerebral hemisphere and from 60 mg of each liver homogenate before drying the extracts under nitrogen gas. We derivatized the fatty acids from the lipid extracts by adding 3 ml of Hilditch reagent and 1.5 ml of methylene chloride to each sample before placing them at 100°C for 1 h. We then neutralized the transmethylation reaction by adding 1 ml of saturated sodium bicarbonate solution to each sample. We performed three hexane washes to isolate the organic phase containing the resulting fatty acid methyl esters (FAMEs). We dried the solvent out of the samples using nitrogen gas, resuspended the FAMEs in 0.5 ml of hexane, and performed sonication. The FAMEs were analyzed by gas chromatography on an Agilent 7890 gas chromatograph with flame ionization detector equipped with a 7693 autosampler. The gas chromatograph column was a ZB wax+ (Phenomenex, USA; 30 m×0.32 mm). We used fatty acid standards (PUFA-1, -3, and Supelco 37 component FAME mix; Sigma-Aldrich, Canada) to identify the fatty acids by retention time. A quantitative standard (cat. no. GLC490, Nu-Chek Prep) was used to check the column every 300 samples to ensure that the areas returned accurately. We added an internal standard (nonadecanoic acid C19:0, Sigma-Aldrich) of known concentration to our samples prior to transmethylation to calculate the concentration of each fatty acid. We analyzed the fatty acid profiles of each cerebral hemisphere separately as part of another project, but we averaged the results of both hemispheres here. Results are expressed as relative concentration using mass percentage of total identified fatty acids.

### Compound-specific isotope analysis

We conducted compound-specific stable isotope analyses on the derivatized n3-PUFAs of the chicks' cerebral hemispheres and livers. The δ^13^C values of the FAMEs of interest were analyzed at the Stable Isotope Laboratory of Memorial University using an Agilent 6890N gas chromatograph coupled via a GC Combustion III interface to a Delta V Plus isotope ratio mass spectrometer (Thermo Scientific). The FAMEs were separated via a BPX70 column (SGE Analytical Science, USA; 50 m×0.32 µm×0.25 µm) with helium (5.0) as the carrier gas with a constant flow rate of 1.5 ml min^−1^. Injector temperature was set at 250°C. The program started at 70°C for 0.6 min before being ramped up at a rate of 10°C min^−1^ until 160°C (hold time 5 min) before reaching 255°C (hold time 2 min) at a rate of 4°C min^−1^. The oxidation reactor consisted of three wires (Cu, Ni and Pt) in a ceramic tube and was held at 940°C, whereas the reduction reactor (three Cu wires in a ceramic tube) was at 600°C.

The samples were injected twice and verified with an isotopically characterized standard (Indiana University fatty acid methyl/ethyl ester mixture F8) analyzed every four samples to correct the measured δ^13^C values of the FAMEs in the samples. The carbon isotope ratios are expressed as parts per thousand (‰) relative to the international standards Vienna Pee Dee Belemnite (VPDB) for δ^13^C following the equation:
(1)

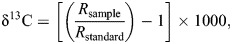
where *R*=^13^C/^12^C.

We corrected for the added methyl group of each FAME by measuring the δ^13^C value of the methanol used for derivatization with an Aurora Total Organic Carbon analyzer, coupled to a Delta Plus XP isotope ratio mass spectrometer [analysis completed by the Ján Veizer (formerly G. G. Hatch) Stable Isotope Laboratory, University of Ottawa]. The methanol δ^13^C values were determined to be −28.21‰, which we used to find the real value of each FAME through the following equation:
(2)

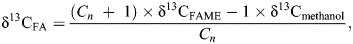
where δ^13^C_FAME_, δ^13^C_FA_ and δ^13^C_methanol_ are the carbon isotopic values of the FAME, the corresponding fatty acid (FA) and the methanol, respectively. *C_n_* corresponds to the number of carbons in the fatty acid chain.

The instruments could only detect FAMES with proportions >1.5% of the total fatty acids detected. Therefore, the only δ^13^C values that could be obtained were those of ALA, EPA and DPA in the tissues of a few chicks and of DHA in all chicks' livers and cerebral hemispheres (Table S1). Given the tracer's failure to enrich the carbon isotopic composition of any n3-PUFAs, in addition to the various n3-PUFAs with levels insufficient to characterize their δ^13^C values in the chicks' tissues, we simply present the DHA-δ^13^C values to inform future researchers about natural differences across colonies.

### Statistical analysis

We performed all statistical analyses in R (version 4.3.2, https://www.r-project.org/). ANOVAs and linear mixed models (LMMs) were validated by ensuring that the residuals were normally distributed and homogeneity of variance was met, as determined using diagnostic Q–Q plots, plots of residuals versus fitted values, and Levene's tests. LMMs were also validated by overlaying the raw data over the simulated responses of the models using histograms. We report effect sizes as partial eta-squared (η_p_^2^) for ANOVAs and as odds ratios for LMMs. For LMMs, we ensured that the distribution of the random effect met the assumption of normality.

#### Flaxseed oil supplementation

We tested whether the experimental chicks supplemented with flaxseed oil incorporated more ALA (mass percentage of total identified fatty acids) into their tissues compared with controls. We used a separate ANOVA model for the liver and brain tissues and included chick colony as a second factor in each model. Both models were initially fitted with an interaction between chick colony and the type of supplement received to account for natural differences in the fatty acid profile of each colony, which could influence the accumulation of the supplemented ALA into their tissues. This interaction term was ultimately dropped after being found to be non-significant for both the liver model and the brain model. We also explored the effects of supplementation on chick mass over the duration of the experiment (72 h) using an LMM that included supplementation treatment (flaxseed oil versus coconut oil) and colony (Kelly's Island versus Salmonier) as fixed factors, age [days post-hatch (dph), 0 to 3] as a continuous predictor, and subject identity as a random effect to account for non-independence among the four body masses recorded for each chick. This LMM also initially included an interaction term between chick colony and the type of supplementation received, but because the term was non-significant, it was dropped and the model was refitted without the interaction.

To test whether nestlings were able to convert the ALA (mass percentage of total identified fatty acids) contained in the flaxseed oil into n3-LCPUFAs, we ran separate ANOVAs for each tissue (liver and brain) and for each ALA derivate (SA, ETA, ETE, EPA, DPA and DHA); treatment group (flaxseed oil versus coconut oil) and colony (Kelly's Island versus Salmonier) were included as factors in each model. The interaction between the factors was never statistically significant and was omitted from the final models. Where SA, ETE and ETA were used as responses, the model residuals did not meet the assumption of normality or of homogeneity of variance. Therefore, we log-transformed (natural log) these responses, which improved the distribution of the residuals of their respective models.

#### Carbon isotopic composition of DHA

We compared the natural carbon isotopic composition of the DHA content of chicks between hatching colonies using an ANOVA for each tissue and controlled for any potential effect of having administered the ALA-^13^C_1_ tracer by including the subject treatment group as a second factor in each model. The final models were fitted without an interaction between chick colony and treatment group because the interaction was consistently found to be non-significant.

## RESULTS

### Flaxseed oil supplementation

**Fig. 3.**
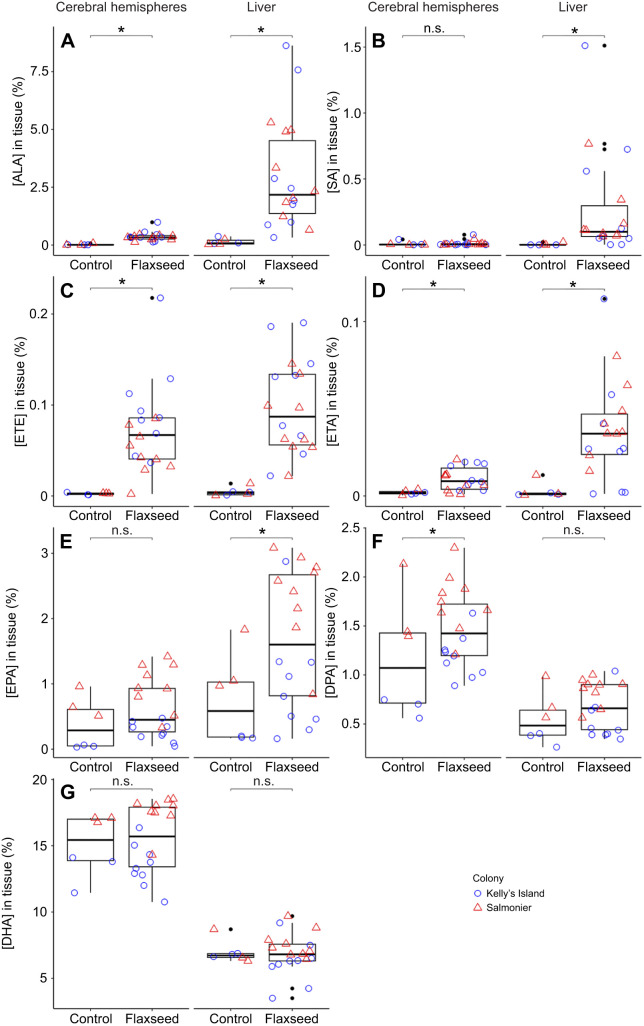
**Levels of omega-3 fatty acid metabolites in the tissues of ring-billed gull chicks following 3** **days of flaxseed oil supplementation (ALA) in comparison to their control counterparts supplemented with coconut oil.** The untransformed concentrations of (A) alpha-linolenic acid (ALA), (B) stearidonic acid (SA), (C) eicosatrienoic acid (ETE), (D) eicosatetraenoic acid (ETA), (E) eicosapentaenoic acid (EPA), (F) docosapentaenoic acid (DPA) and (G) docosahexaenoic acid (DHA) in the chicks' tissues are expressed as relative concentration (mass percentage of total identified fatty acids). Raw data points are colored and shaped based on chick hatching colony: Kelly's Island, blue circles (*N*=12 chicks); Salmonier, red triangles (*N*=12 chicks). *Significant differences between treatment groups; n.s., not significant.

Chicks supplemented with flaxseed oil (57% ALA, 0% n3-LCPUFAs; Table 1) incorporated more ALA into their livers and cerebral hemispheres as compared with control chicks that were supplemented with coconut oil (0% ALA, 0% n3-LCPUFAs; Table 1); hatching colony did not influence ALA levels in the liver or cerebral hemispheres (Fig. 3A, Table 2; Table S2).

**Table 2.** **Effect of the flaxseed oil treatment on the levels of each omega-3 fatty acid in ring-billed gull hatchling tissues after 3** **days of supplementation**

Response	*R* ^2^_adj_	Factor	d.f.	SS	*F*	*P*	η_p_^2^
Liver							
ALA	0.21	Treatment	1	36.77	8.07	0.010*	0.278
		Colony	1	0.04	0.01	0.922	<0.001
		Residuals	21	95.63			
log(SA)	0.55	Treatment	1	73.31	28.94	<0.001*	0.564
		Colony	1	3.38	1.33	0.261	0.026
		Residuals	21	53.19			
log(ETE)	0.78	Treatment	1	53.15	81.37	<0.001*	0.792
		Colony	1	0.26	0.40	0.536	0.004
		Residuals	21	13.72			
log(ETA)	0.53	Treatment	1	34.66	24.48	<0.001*	0.503
		Colony	1	4.49	3.17	0.089	0.065
		Residuals	21	29.73			
EPA	0.55	Treatment	1	4.04	8.59	0.008*	0.167
		Colony	1	10.33	21.98	<0.001*	0.426
		Residuals	21	9.87			
DPA	0.48	Treatment	1	0.09	2.75	0.112	0.063
		Colony	1	0.65	20.30	<0.001*	0.461
		Residuals	21	0.67			
DHA	0.11	Treatment	1	0.04	0.02	0.878	<0.001
		Colony	1	8.33	4.92	0.038*	0.190
		Residuals	21	35.57			
Cerebral hemispheres							
ALA	0.416	Treatment	1	0.53	17.71	<0.001*	0.450
		Colony	1	0.02	0.68	0.421	0.017
		Residuals	21	0.62			
log(SA)	−0.05	Treatment	1	0.45	0.29	0.598	0.013
		Colony	1	1.08	0.68	0.417	0.031
		Residuals	21	33.24			
log(ETE)		Treatment	1	45.13	63.65	<0.001*	0.728
	0.74	Colony	1	1.95	2.75	0.112	0.031
		Residuals	21	14.89			
log(ETA)	0.35	Treatment	1	10.84	13.32	0.001*	0.377
		Colony	1	0.85	1.04	0.319	0.029
		Residuals	21	17.09			
EPA	0.67	Treatment	1	0.25	3.88	0.062	0.057
		Colony	1	2.82	43.70	<0.001*	0.637
		Residuals	21	1.36			
DPA	0.60	Treatment	1	0.42	5.06	0.035*	0.087
		Colony	1	2.65	32.00	<0.001*	0.551
		Residuals	21	1.74			
DHA	0.69	Treatment	1	0.94	0.50	0.486	0.007
		Colony	1	96.70	51.47	<0.001*	0.705
		Residuals	21	39.46			

Relative concentrations (mass percentage of total identified fatty acids) of stearidonic acid (SA), eicosatrienoic acid (ETE), eicosatetraenoic acid (ETA), eicosapentaenoic acid (EPA), docosapentaenoic acid (DPA) and docosahexaenoic acid (DHA) were quantified in the chicks' livers and cerebral hemispheres. Hatching colony was included in each ANOVA model to control for natural differences in the tissues. *Significant differences. SA, ETE and ETA responses were log-transformed for both types of tissues to meet ANOVA assumptions of normality and homogeneity of variance.

The hatchlings gained mass as they aged over the 72 h in captivity (LMM: Likelihood ratio χ^2^_1_=138.26, *P*<0.001; odds ratios=35.41, 95% CI=19.54, 64.17; Fig. S1). The Salmonier chicks were heavier than those collected at the Kelly's Island colony (LMM: LR χ^2^_1_=14.98, *P*<0.001; odds ratios=163.07, 95% CI=12.34, 2154.19; Fig. S1), but hatchling mass was not influenced by whether they received the ALA-^13^C_1_ tracer and the flaxseed oil supplement or the coconut oil (LMM: LR χ^2^_1_=0.63, *P*=0.425; odds ratios=0.30, 95% CI=0.01, 13.43; Fig. S1).

Chicks hatched at the Salmonier colony had more EPA, DPA and DHA in both their cerebral hemispheres and their livers compared with nestlings hatched at Kelly's Island (Fig. 3E–G, Table 2; Table S2). In hepatic tissues, Salmonier nestlings presented EPA, DPA and DHA levels (mean±s.d.) of 2.10±0.76%, 0.81±0.15% and 7.50±1.03%, respectively. Meanwhile, chicks from Kelly's Island showed levels of 0.79±0.76% for EPA, 0.48±0.20% for DPA and 6.32±1.38% for DHA. Similarly, for Kelly's Island chicks, the EPA, DPA and DHA composition of their cerebral tissues amounted to 0.21±0.15%, 1.06±0.29% and 13.38±1.48%, respectively, while reaching 0.90±0.33% (EPA), 1.72±0.31% (DPA) and 17.40±1.08% (DHA) for Salmonier nestlings. In contrast, the levels of SA, ETE and ETA did not differ significantly between colonies for both tissues (Fig. 3B–D, Table 2, Table S2).

After controlling for the effect of colony, experimental chicks that received the flaxseed oil supplement showed greater concentrations of SA, ETE, ETA and EPA in their liver compared to control chicks that received coconut oil; the levels of DPA and DHA in the liver did not differ significantly between experimental and control chicks (Fig. 3, Table 2; Table S2). ETE, ETA and DPA levels in the cerebral hemispheres were also higher in the experimental nestlings that received flaxseed oil than in control chicks that received coconut oil (Fig. 3C,D,F, Table 2; Table S2). The cerebral levels of SA, EPA and DHA did not differ significantly between treatment groups (Fig. 3B,E,G, Table 2; Table S2).

### Carbon isotopic composition of DHA

**Fig. 4.**
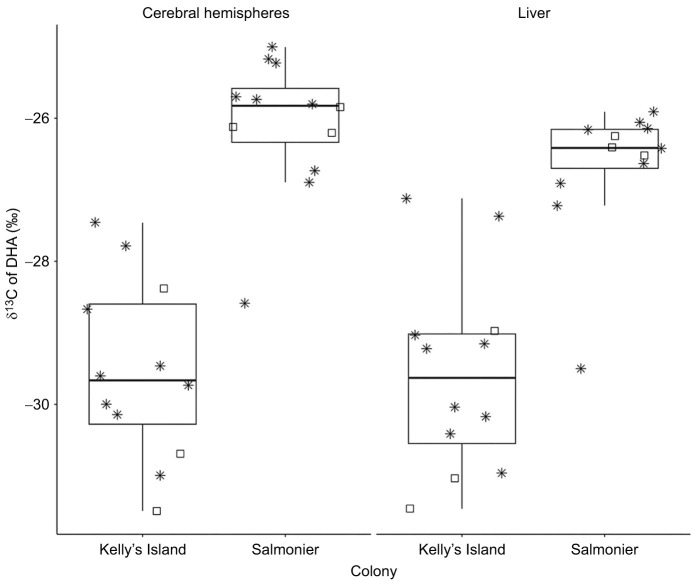
**Carbon isotope composition (δ^13^C; ‰) of docosahexaenoic acid (DHA) detected in the tissues of ring-billed gull chicks based on their hatching colony (Kelly's Island, urban colony; Salmonier, rural colony).** Raw data are represented by the shapes and indicate the supplementation groups (square, control group; star, alpha-linolenic acid carbon isotopic tracer group).

When considering the δ^13^C values of hepatic DHA, chicks differed significantly based on their hatching colony, with Salmonier birds showing greater carbon enrichment than their Kelly's Island counterparts (mean difference of 2.90‰, 95% CI=1.89, 3.91; Fig. 4, Table 3; Table S1). The same results were obtained in the cerebral hemispheres, where colony was a significant predictor of δ^13^C values; Salmonier chicks had an enriched ^13^C profile associated with their cerebral DHA compared with the Kelly's Island nestlings (mean difference of 3.45‰, 95% CI=2.49, 4.40; Fig. 4, Table 3; Table S1). Supplementation with the ALA-^13^C_1_ tracer or with the control did not influence the DHA δ^13^C values of the liver or cerebral hemispheres of the subjects (Fig. 4, Table 3; Table S1).

**Table 3.** Carbon isotope composition (δ^13^C; ‰) of docosahexaenoic acid (DHA) stored in the livers and cerebral hemispheres of ring-billed gull chicks based on their hatching colony being located either in a rural environment (Salmonier) or in an urban environment (Kelly's Island)

Response	*R* ^2^_adj_	Factors	d.f.	SS	*F*	*P*	η_p_^2^
Liver							
DHA-δ^13^C	0.60	Colony	1	50.46	35.17	<0.001*	0.620
		Treatment	1	0.78	0.54	0.469	0.010
		Residuals	21	30.12			
Cerebral hemispheres							
DHA-δ^13^C	0.70	Colony	1	71.28	55.28	<0.001*	0.719
		Treatment	1	0.78	0.60	0.446	0.008
		Residuals	21	27.08			

Treatment groups (received the alpha-linolenic acid carbon isotopic tracer or not) were included in each ANOVA model to control for the possibility that the tracer influenced the carbon isotopic value of the chicks' DHA. *Significant differences.

## DISCUSSION

We provide the first evidence that the chick of a generalist seabird, the ring-billed gull, can bioconvert ALA into its omega-3 derivates SA, ETE and ETA, in addition to showing some abilities to biosynthesize the n3-LCPUFAs EPA and DPA. Furthermore, we show that these results are consistent across two colonies of wild birds despite pronounced natural differences in the n3-LCPUFA profiles of their tissues. We also show that the natural δ^13^C values of DHA differed between colonies. Unfortunately, we failed to detect the presence of the isotopic tracer in the chicks' tissues such that we could not ascertain the metabolic fate of dietary ALA. Whereas ring-billed gull chicks appeared to lack proficiency at bioconverting ALA all the way to DHA based on the flaxseed oil supplementation experiment, further studies using isotopic labels or exploring the gene expression of elongases and desaturases are necessary to show a complete inability to biosynthesize DHA.

Hatchlings fed flaxseed oil rich in ALA showed increased levels of the n3-PUFA intermediates SA, ETE and ETA in their liver and ETE and ETA in their cerebral hemispheres, as compared with control chicks that were only fed the coconut oil. These experimental chicks also bioconverted ALA into some n3-LCPUFAs, as evidenced by elevated levels of EPA in their livers and DPA in their cerebral hemispheres. This indicates that ring-billed gulls exhibit elongase-5 and elongase-2 activity in their tissues, as is common across chordates (Monroig et al., 2016), in addition to expressing some levels of the Δ5- and Δ6- desaturases. In contrast, several other predators, particularly marine predators, do not express the genes for Δ5- or Δ6-desaturases, rendering them incapable of producing some or all n3-LCPUFAs from ALA (Fig. 1). For example, no Δ5-desaturase activity has been found in marine carnivorous teleosts including barramundi (*Lates calcarifer*; Tu et al., 2012), red seabream (*Pagrus major*) and Japanese flounder (*Paralichthys olivaceus*; Nyunoya et al., 2021). For other predators, an absence of Δ6-desaturase activity prevents the bioconversion of ALA into its longer-chain derivates (cats; Trevizan et al., 2012), whereas certain animals are impacted equally by the poor expression of both Δ5- and Δ6- desaturases (Atlantic cod, *Gadus morhua*: Agaba et al., 2005; Tocher et al., 2006; pompano, *Trachinotus ovatus*: Wang et al., 2020a).

Although our subjects appear to express all enzymes of the Sprecher pathway, we did not find evidence of supplemental ALA ultimately being bioconverted into DHA despite finding evidence of endogenous biosynthesis for all other ALA derivates. The lack of DHA synthesis could be explained by a bottleneck effect caused by an excess of ALA, an issue common in vertebrates that favour the Sprecher pathway (Monroig et al., 2016; Twining et al., 2021a). The Δ6-desaturase route is rate-limiting because Δ6-desaturase has multiple substrates. The short-chain omega-6 LA is one such substrate and high levels of LA in animal tissues tend to saturate the binding sites of Δ6-desaturase, effectively inhibiting the binding and, thereby, conversion of ALA (El-Zenary et al., 2020; Sprecher et al., 1995; Wood et al., 2015). Nonetheless, ALA is the substrate which holds by far the greatest binding affinity (Gregory et al., 2011; Holman, 1998). As such, high ALA levels in tissues can compromise the ability of other substrates, including 24:5n3 (the immediate derivate of DPA in the Sprecher pathway; Fig. 1), to bind with Δ6-desaturase, effectively preventing their ultimate conversion into DHA (Sprecher, 2002; Yamazaki et al., 1992; Zheng et al., 2004). Ring-billed gulls are now one of several species that have been shown to convert ALA into EPA and DPA, possibly without increasing DHA [e.g. guinea pigs (*Cavia porcellus*): Fu and Sinclair, 2000; turkeys (*Meleagris gallopavo domesticus*): Jankowski et al., 2012; piglets (*Sus domesticus*): Tanghe et al., 2015; rainbow trout (*Oncorhynchus mykiss*): Turchini and Francis, 2009]. Even some herbivores such as cockatiels (*Nymphicus hollandicus*) do not increase their tissue levels of DHA when fed a flaxseed oil supplement, despite successfully increasing their levels of EPA and DPA (Heinze et al., 2012). Failure to synthesize DHA from ALA despite expressing all the required enzymes is a taxonomically widespread phenomenon that may be explained by species favouring the rate-limiting Δ6-desaturase pathway combined with an insufficient expression of the elongases and desaturases that define this pathway (Cartoni Mancinelli et al., 2022). Because our tracer study failed to track the fate of the isotopically labelled ALA in the chicks' tissues, further research using compound-specific stable isotope analysis is required to ascertain whether ring-billed gulls are completely unable to biosynthesize DHA from ALA, as opposed to being inefficient at this process. We also cannot discount the possibility that DHA synthesis did occur but was used to maintain current levels without accreting further, thereby remaining undetectable without a highly labelled tracer. However, this seems unlikely based on our previous studies showing that ring-billed gull chicks and adults accrete DHA in their cerebral tissues when it is available in their diets, often to levels exceeding those seen in the present study (Lamarre et al., 2021; Lamarre and Wilson, 2024).

Among freshwater vertebrates, the upregulation of the genes coding for these key desaturases (Δ5 and Δ6) and elongases (5 and 2) is widespread, which suggests that maintaining high tissue levels of n3-LCPUFAs is critical for these animals (Hazel and Williams, 1990; Matsushita et al., 2020; Twining et al., 2021a). In particular, fish that are either of freshwater origin or that have successfully diverged from their marine ancestors to colonize freshwater habitats tend to express those genes at high levels, which facilitates rapid bioconversion of ALA into DHA (Ishikawa et al., 2019; Karapanagiotidis et al., 2007; Matsushita et al., 2020; Tocher, 2010). Efficiently converting ALA into DHA also has been demonstrated in domestic ducks (Juodka et al., 2022; Shahid et al., 2019; Wang et al., 2022) and caimans (Leiva et al., 2021). Although many populations of ring-billed gulls primarily exploit freshwater and terrestrial habitats (Pollet et al., 2012), their ALA bioconversion abilities appear to be more limited than those of aquatic species such as ducks. Instead, ring-billed gulls, which are members of the diverse order Charadriiformes that includes shorebirds, gulls and auks, more closely resemble another charadriiform, the western sandpiper, a shorebird that feeds on benthic invertebrates at the tide line (Franks et al., 2020). Like ring-billed gulls, western sandpipers show limited ability to bioconvert ALA into EPA and no evidence of being capable of furthering the conversion to DHA (Dick et al., 2024).

Isotopically labelled tracer studies are the gold standard for tracking the metabolic fate of ALA in animal tissues (Ali et al., 2025; Whiteman et al., 2019). Unfortunately, our methodology failed to enrich the carbon isotopic values of any n3-PUFA such that it was not possible to ascertain whether the increased levels of the ALA derivates detected here stemmed directly from the ALA tracer administered. This is likely explained by the tracer being insufficiently enriched because only one carbon out of its 18-carbon chain was labelled with the ^13^C_1_ tracer; in contrast, other studies used heavily enriched tracers in which all 18 carbons were labelled (e.g. Twining et al., 2018, 2021b). We also cannot rule out that the tracer was simply not absorbed by the hatchlings or that the concentration given was too low to be detectable. Although future studies should take into account these methodological considerations, the expensive nature of using large amounts of heavily labelled tracers is a well-known constraint. Instead, other authors have discussed the possibility of using the natural δ^13^C values of dietary fatty acids arising from distinct ecosystems (e.g. terrestrial versus marine) to follow their fate in animal tissues, preventing the need to rely on costly tracers (Giuliano et al., 2018; Lacombe and Bazinet, 2021).

Nonetheless, because the DHA present in the chicks' tissues could only have come from the yolk, their δ^13^C values were informative about their natural provenance (Meier-Augenstein, 2002). Compared with chicks from Kelly's Island, the DHA in the livers and brains of chicks from Salmonier had enriched carbon values. This aligns with the distinct fatty acid profiles of each colony, in addition to paralleling the isotopic profiles of the adults nesting at those colonies. Adults nesting at Salmonier are marine foragers and show enriched ^13^C values in their tissues (red blood cells mean±s.d. in 2020: −20.09±0.74‰, in 2021: −19.95±0.58‰), compared with adults nesting at Kelly's Island (red blood cells mean±s.d. in 2020: −22.98±0.71‰, in 2021: −22.81±0.84‰) and foraging on anthropogenic resources depleted in carbon (Lamarre et al., 2022; Lamarre and Wilson, 2024). Ring-billed gulls tend to return to their nesting grounds at least 3 weeks ahead of laying (Giroux et al., 2016), and egg formation is thought to last for fewer than 12 days before the females lay (Bolton et al., 1992; Brown, 1967; Roudybush et al., 1979). As a result, the nutrient composition of their yolk tends to reflect the diet of females during egg formation (Blount et al., 2002; Indykiewicz et al., 2023). The greater carbon enrichment of the DHA in the tissues of chicks from Salmonier, in addition to their overall greater levels of hepatic and encephalic n3-LCPUFAs, corroborates the findings of prior studies indicating that Salmonier females primarily exploit nutrients of marine origin (Lamarre et al., 2022; Lamarre and Wilson, 2024) and transfer them into their yolk, and ultimately into the tissues of the developing embryos (Hobson et al., 1997; Serré et al., 2022). In contrast, females nesting at Kelly's Island are known to consume a much more anthropogenic and terrestrial diet (Lamarre et al., 2022; Lamarre and Wilson, 2024), which was seemingly reflected in their chicks based on the lower levels of n3-LCPUFAs and the carbon-depleted DHA values found in their tissues (Hixson et al., 2014; Lacombe et al., 2017; Serré et al., 2022). Nonetheless, because the parents' tissues were not collected as part of this study, we cannot rule out whether the difference in the DHA-δ^13^C values of the chicks' tissues was also influenced by physiological differences specific to each colony, such as possible maternal or *in ovo* synthesis of DHA. Future studies should investigate the DHA-δ^13^C values of the undeveloped yolk to establish whether the DHA stored in the hatchlings' tissues shows similar values, suggesting a simple transfer during embryogenesis. In contrast, comparatively depleted δ^13^C values would suggest that some of their DHA arose from bioconversion *in ovo* (Pedersen et al., 2025). The large natural difference in n3-LCPUFAs between the two colonies did not appear to affect bioconversion rates because the interaction between colony and supplementation treatment was not statistically significant for any n3-PUFAs. However, we must caution that our sample size might have been too small to accurately detect the impact of the chicks' natural levels of n3-LCPUFAs on their bioconversion abilities. Nonetheless, because metabolic demands for DHA are at their peak during early life to sustain rapid brain development (Price et al., 2018; Speake and Wood, 2005), we expected the bioconversion abilities of chicks to be at their maximum regardless of the colony of origin, especially because the diet provided in captivity was devoid of preformed n3-LCPUFAs.

Our finding that ring-billed gull (suborder Lari) chicks appear not to biosynthesize DHA, together with previous research showing that western sandpipers (suborder Scolopaci) show the same limitation (Dick et al., 2024), suggests that Charadriiformes in general may depend on dietary sources of DHA. Determining whether all charadriiform, and perhaps all marine-dwelling, birds are incapable of synthesizing DHA should be a priority for future research as it would provide insight into the potential fitness consequences of an increasingly anthropogenic diet. Although many species and populations of gulls are already known to forage primarily on anthropogenic food lacking preformed n3-LCPUFAs (e.g. Lamarre et al., 2021; Seif et al., 2018; Weiser and Powell, 2010), other marine-dwelling birds considered as fish specialists now also rely on anthropogenic resources with impoverished n3-LCPUFAs profiles. For example, when lipid-rich pelagic fish are less abundant owing to warming waters or overfishing, gannets (*Morus* spp.) feed on lipid-poor groundfish offal (Grémillet et al., 2008; Votier et al., 2013), which would severely reduce their intake of preformed n3-LCPUFAs. Similar dietary shifts have been observed in shearwaters and fulmars (Procellariidae; Maynard et al., 2020). Lower levels of n3-LCPUFAs in the brain are associated with reduced membrane flexibility and increased risk of membrane damage (DiNicolantonio and O'Keefe, 2020). Maintaining high levels of cerebral DHA throughout one's life protects the brain's function, mitigates brain senescence and supports memory formation (Kim et al., 2014; Xia et al., 2023). Long-lived seabirds relying on anthropogenic resources deficient in n3-LCPUFAs might be at risk of cognitive decline if, like ring-billed gull nestlings, they show poor ability to endogenously synthesize DHA.

## Supplementary Material

10.1242/jexbio.250429_sup1 Supplementary information
