# Supplementary material for: Biosynthesis of long-chain omega-3 fatty acids by the nestlings of a generalist seabird
Source: J Exp Biol. 2025 Dec 2;228(23):jeb250429. doi: 10.1242/jeb.250429 (PMC12745934; doi:10.1242/jeb.250429)
Supplement: Supplementary information [file jexbio-228-250429-s1.pdf]

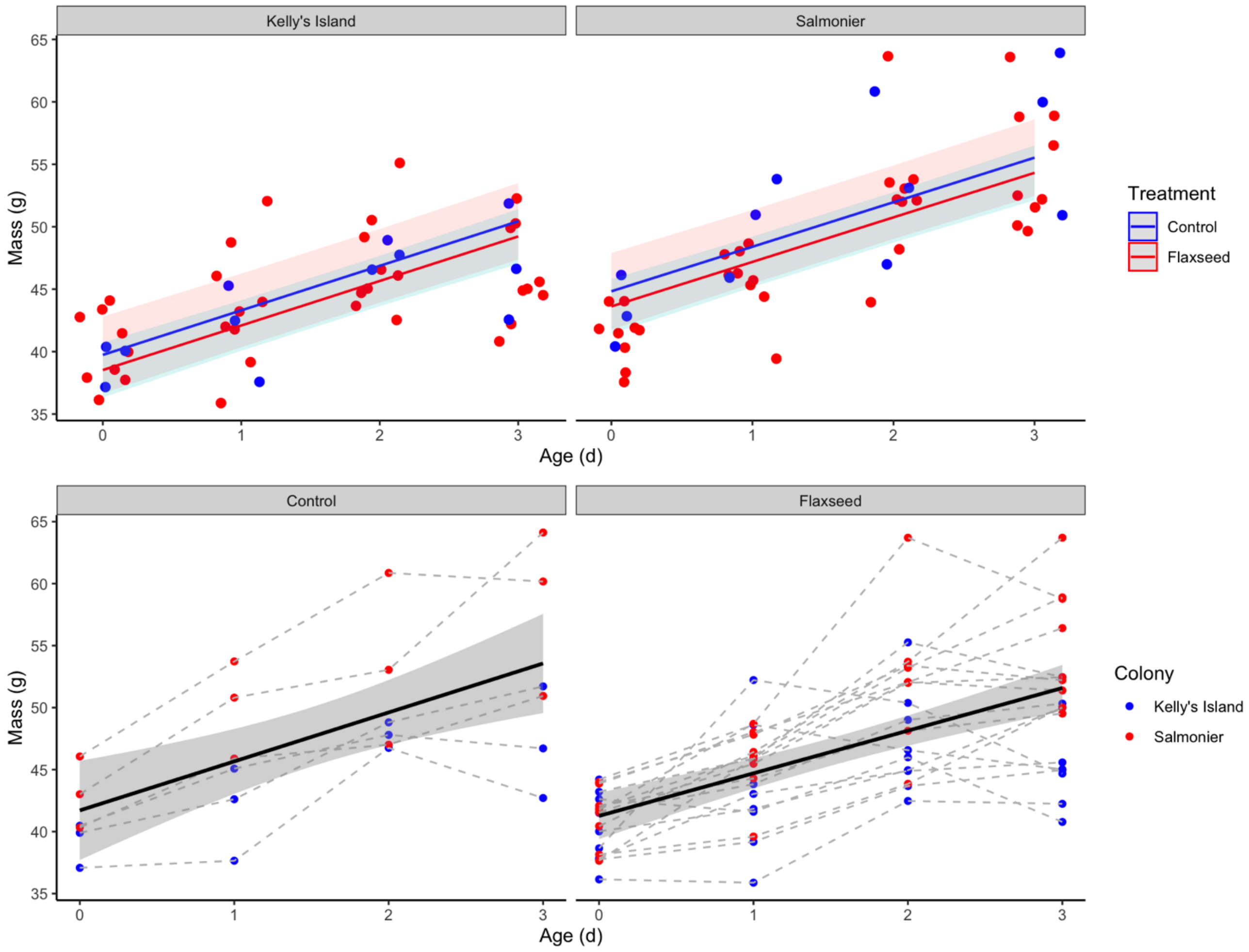

Fig. S1. Body mass (g) trajectory of ring-billed gull hatchlings from hatch (collection day as day 0) unti 3 days post-hatch based on whether they received the flaxseed oil supplementation or the caloric control that was devoid of omega-3s. A) The relationships (± 95% CI) between the chicks' mass over time, based on their supplementation group and colony and as predicted by our mixed model, are represented by the straight lines with their respective shading. The raw data are represented by the points, which have been jittered horizontally to facilitate viewing. B) Individual body mass trajectories of each chick presented with the overall trendline for each supplementation treatment group and differentiated by colony.

Journal of Experimental Biology • Supplementary information

Table S1. Carbon isotope values ($\delta^{13}C$ (‰)) of the main omega-3 polyunsaturated fatty acids (alpha-linolenic acid (ALA), eicosapentaenoic acid (EPA), docosapentaenoic acid (DPA), and docosahexaenoic acid (DHA)) detected in the tissues of ring-billed gull hatchlings fed 1.36 mg of ALA-$^{13}C_1$ tracer dissolved in 100 uL of coconut oil or 100 uL of coconut oil without the tracer (control). The sample sizes indicate the number of subjects for which each fatty acid's $\delta^{13}C$ values could be quantified out of a maximum of N=3 for each control group and N=9 for each ALA-$^{13}C_1$ group.

		Kelly's Island				Salmonier			
		Cerebral hemispheres		Liver		Cerebral hemispheres		Liver	
		Control	ALA-$^{13}C_1$	Control	ALA-$^{13}C_1$	Control	ALA-$^{13}C_1$	Control	ALA-$^{13}C_1$
ALA	N	NA	NA	NA	5	NA	NA	NA	7
	Mean (SD)	NA	NA	NA	-32.84 (1.66)	NA	NA	NA	-31.53 (1.19)
EPA	N	NA	NA	NA	2	NA	1	1	8
	Mean (SD)	NA	NA	NA	-31.54 (2.10)	NA	-28.76 (0.0)	-31.91 (0.0)	-30.58 (0.73)
DPA	N	NA	NA	NA	NA	1	NA	1	NA
	Mean (SD)	NA	NA	NA	NA	-29.02 (0.0)	NA	-29.19 (0.0)	NA
DHA	N	3	9	3	9	3	9	3	9
	Mean (SD)	-30.19 (1.32)	-29.32 (1.08)	-30.49 (1.09)	-29.27 (1.24)	-26.06 (0.15)	-26.10 (1.08)	-26.40 (0.11)	-26.77 (1.05)

NA indicates instances where the $\delta^{13}C$ values could not be determined because the fatty acid levels were too low in all subjects that were part of the given group. The $\delta^{13}C$ values of the ALA derivates SA, ETA, ETE could not be quantified in either tissue due to insufficient concentrations.

Table S2. Fatty acid composition, expressed as the percentage of total identified fatty acids, of the tissues of ring-billed gull hatchlings after receiving a flaxseed oil supplement or a caloric equivalent devoid of omega-3s (coconut oil) from 1 day post hatch until 3 days post hatch.

	Kelly's Island				Salmonier			
	Cerebral hemispheres		Liver		Cerebral hemispheres		Liver	
	Control	Flaxseed	Control	Flaxseed	Control	Flaxseed	Control	Flaxseed
Fatty acid (%)	N = 3	N = 9	N = 3	N = 9	N = 3	N = 9	N = 3	N = 9
C14:0	0.71±0.13	0.65±0.18	0.85±0.56	2.44±2.62	0.73±0.07	0.53±0.14	3.66±1.98	3.08±1.19
C14:1	0.80±0.09	0.83±0.07	Trace	0.02±0.01	0.78±0.07	0.78±0.05	0.07±0.04	0.04±0.03
C16:0	30.78±1.21	31.96±1.09	7.76±0.88	9.43±1.99	30.66±0.58	31.29±1.11	13.96±3.15	11.79±2.39
C16:1*n*−11	0.06±0.01	0.05±0.01	Trace	Trace	0.09±0.01	0.08±0.02	0.01±0.01	Trace
C16:1*n*−9	0.86±0.12	0.76±0.16	0.18±0.02	0.18±0.03	0.98±0.1	0.76±0.05	0.39±0.11	0.23±0.05
C16:1*n*−7	1.13±0.12	1.04±0.13	0.30±0.05	0.24±0.06	1.36±0.07	1.16±0.09	0.90±0.19	0.63±0.20
C16:1*n*−5	0.80±0.04	0.83±0.07	Trace	0.05±0.02	0.90±0.10	0.85±0.06	0.06±0.01	0.05±0.01
C16:2*n*−6	0.07±0.02	0.06±0.02	0.14±0.03	0.12±0.05	Trace	0.02±0.01	Trace	0.09±0.04
C17:0	0.19±0.02	0.20±0.04	0.17±0.01	0.21±0.05	0.19±0.03	0.18±0.03	0.15±0.03	0.18±0.04
C16:3*n*−4	0.20±0.02	0.22±0.04	0.19±0.02	0.19±0.07	0.24±0.03	0.23±0.02	0.24±0.03	0.19±0.06
C16:4*n*−3	0.76±0.10	0.66±0.09	0.11±0.01	0.10±0.03	0.79±0.12	0.81±0.12	0.06±0.01	0.08±0.02
C16:4*n*−1	0.18±0.01	0.15±0.03	Trace	0.04±0.05	0.15±0.03	0.15±0.03	Trace	0.03±0.01
C18:0	17.09±0.27	17.13±0.46	11.33±0.5	13.45±3.94	17.77±0.28	17.44±0.54	10.99±1.99	13.09±1.86
C18:1*n*−11	2.00±2.71	3.43±3.02	Trace	0.03±0.03	0.09±0.02	1.40±2.57	0.06±0.01	0.05±0.03
C18:1*n*−9	11.42±2.45	9.75±2.03	55.82±1.33	44.81±7.85	12.92±0.43	11.49±1.83	46.01±2.63	43.24±8.28
C18:1*n*−7	3.11±0.78	2.47±0.83	0.83±0.08	0.98±0.14	4.45±0.13	3.78±0.76	5.22±5.55	1.26±0.21
C18:1*n*−6	0.04±0.04	0.02±0.02	Trace	0.02±0.01	Trace	0.02±0.02	0.02±0.01	0.03±0.04
C18:1*n*−5	0.02±0.01	0.02±0.02	Trace	0.01±0.02	0.02±0.01	0.01±0.01	0.05±0.03	0.03±0.01
C18:2*n*−6 (LA)	0.39±0.1	0.84±0.14	2.27±0.23	4.71±1.11	0.42±0.1	0.75±0.11	2.27±0.48	4.60±1.14
C18:2*n*−4	Trace	Trace	0.11±0.02	0.08±0.04	0.05±0.01	0.04±0.01	0.06±0.01	0.05±0.02
C18:3*n*−4	0.01±0.01	0.01±0.01	0.05±0.05	0.10±0.06	Trace	Trace	0.05±0.04	0.05±0.04
C18:3*n*−3 (ALA)	Trace	0.40±0.24	0.18±0.14	3.04±2.81	0.03±0.03	0.32±0.1	0.10±0.10	2.95±1.64
C18:4*n*−3 (SA)	0.02±0.02	0.01±0.02	Trace	0.34±0.34	Trace	0.01±0.01	0.01±0.01	0.20±0.20
C18:4*n*−1	0.02±0.02	0.01±0.01	Trace	Trace	Trace	0.01±0.01	Trace	Trace
C20:0	0.05±0.02	0.05±0.02	0.11±0.03	0.12±0.02	0.06±0.01	0.05±0.01	0.12±0.04	0.13±0.02
C20:1*n*−11	0.12±0.07	0.13±0.06	0.13±0.01	0.09±0.05	0.16±0.06	0.20±0.05	0.15±0.05	0.14±0.05

Journal of Experimental Biology • Supplementary information

C20:1*n*−9	0.20±0.10	0.10±0.05	0.21±0.05	0.19±0.04	0.19±0.07	Trace	0.25±0.09	0.27±0.09
C20:1*n*−7	Trace	0.03±0.05	0.02±0.01	0.01±0.01	0.02±0.02	0.01±0.01	0.08±0.02	0.05±0.02
C20:2*n*−6	0.94±0.41	0.26±0.13	0.07±0.01	0.13±0.04	1.69±0.41	0.71±0.32	Trace	0.08±0.02
C20:4*n*−6 (AA)	9.31±0.32	9.15±0.52	9.45±0.58	9.57±2.73	5.02±0.80	5.60±1.08	4.78±1.57	5.91±1.34
C20:3*n*−3 (ETE)	Trace	0.10±0.05	Trace	0.11±0.06	Trace	0.05±0.02	0.01±0.01	0.08±0.04
C20:4*n*−3 (ETA)	Trace	0.01±0.01	Trace	0.03±0.03	Trace	0.01±0.01	Trace	0.04±0.02
C20:5*n*−3 (EPA)	0.05±0.01	0.26±0.14	0.18±0.01	0.99±0.78	0.70±0.19	0.96±0.35	1.28±0.39	2.37±0.65
C22:0	0.04±0.02	0.03±0.01	0.03±0.02	0.04±0.02	0.02±0.01	0.02±0.01	0.03±0.02	0.04±0.02
C22:1*n*−9	0.01±0.01	0.01±0.01	Trace	Trace	Trace	0.01±0.01	Trace	Trace
C22:1*n*−7	Trace	Trace	Trace	Trace	Trace	Trace	Trace	Trace
C22:2*n*−6	0.13±0.11	0.03±0.03	0.01±0.01	Trace	0.10±0.06	0.03±0.03	0.01±0.01	0.01±0.01
C22:4*n*−6	1.79±0.10	1.64±0.13	0.63±0.04	0.48±0.10	0.42±0.16	0.60±0.31	0.21±0.05	0.22±0.08
C22:3*n*−3	Trace	Trace	Trace	Trace	0.03±0.04	Trace	Trace	Trace
C22:5*n*−6	2.77±0.27	1.88±0.49	1.48±0.07	0.80±0.43	0.13±0.06	0.18±0.21	0.16±0.11	0.15±0.05
C22:4*n*−3	Trace	Trace	Trace	Trace	Trace	Trace	Trace	Trace
C22:5*n*−3 (DPA)	0.67±0.08	1.19±0.21	0.35±0.06	0.53±0.21	1.66±0.34	1.75±0.29	0.74±0.18	0.84±0.13
C24:0	Trace	0.02±0.02	0.03±0.01	0.01±0.01	0.01±0.01	0.01±0.01	0.03±0.02	0.01±0.02
C22:6*n*−3 (DHA)	13.12±1.18	13.47±1.56	6.77±0.10	6.17±1.56	16.98±0.15	17.54±1.21	7.18±1.07	7.60±1.00
C24:1	0.02±0.01	0.02±0.01	Trace	0.02±0.01	0.02±0.01	0.01±0.01	0.03±0.01	0.04±0.01
Σ SFAs[a]	48.89±0.82	50.04±1.13	20.29±1.81	25.71±5.11	49.45±0.60	49.52±0.95	28.93±6.22	28.33±4.64
Σ MUFAs[b]	20.59±0.92	19.49±0.68	57.66±1.19	46.68±7.87	21.99±0.27	20.60±0.27	53.32±7.98	46.06±8.16
Σ PUFAs[c]	30.44±1.56	30.39±1.54	22.02±0.63	27.55±3.84	28.44±0.67	29.77±0.96	17.69±2.86	25.55±4.00
Σ *n*−6 FAs[d]	15.2±0.26	13.78±0.96	13.89±0.83	15.69±2.90	7.68±1.09	7.84±1.46	7.81±1.95	10.97±2.09
Σ *n*−3 FAs[e]	13.87±1.29	15.45±1.78	7.49±0.20	11.22±2.7	19.37±0.41	20.63±1.82	9.33±0.95	14.09±2.56
Σ *n*−3 LC FAs[f]	13.83±1.28	14.93±1.59	7.31±0.08	7.69±1.86	19.34±0.38	20.25±1.75	9.21±0.95	10.81±1.56

[a] Sum of saturated fatty acids: C14:0+C16:0+C17:0+C18:0+C20:0+C22:0+C24:0

[b] Sum of monounsaturated fatty acids: C14:1+C16:1n−11+C16:1n−9+C16:1n−7+C16:1n−5+C18:1n−11+C18:1n−9+C18:1n−7+C18:1n−6+C18:1n−5+C20:1n−11+C20:1n−9+C20:1n−7+C22:1n−9+C22:1n−7+ C24:1

[c] Sum of polyunsaturated fatty acids: C16:2n−6+C16:3n−4+C16:4n−3+C16:4n−1+C18:2n−6+C18:2n−4+C18:3n−4+C18:3n−3+C18:4n−3+C18:4n−1+C20:2n−6+C20:4n−6+C20:3n−3+C20:4n−3+C20:5n−3+C22:2n−6+C22:4n−6+C22:3n−3+C22:5n−6+C22:4n−3+C22:5n−3+C22:6n−3

[d] Sum of omega-6 polyunsaturated fatty acids: C18:2n−6+C20:2n−6+C20:4n−6+C22:4n−6+C22:5n−6

[e] Sum of omega-3 polyunsaturated fatty acids: C18:3n−3+C18:4n−3+C20:3n−3+C20:4n−3+C20:5n−3+C22:5n−3+C22:6n−3

[f] Sum of omega-3 long chain polyunsaturated fatty acids: C20:5n−3+C22:5n−3+C22:6n−3

Trace: Fatty acid found to be below 0.01%

Journal of Experimental Biology • Supplementary information
